# Happy to breed in the city? Urban food resources limit reproductive output in Western Jackdaws

**DOI:** 10.1002/ece3.2733

**Published:** 2017-02-01

**Authors:** Eva Meyrier, Lukas Jenni, Yves Bötsch, Stephan Strebel, Bruno Erne, Zulima Tablado

**Affiliations:** ^1^ Swiss Ornithological InstituteSempachSwitzerland; ^2^ Büro für Landschaftspflege & FaunistikMosimann & StrebelInsSwitzerland; ^3^ Workshops for Scientific Support and Equipment of the University of KonstanzKonstanzGermany

**Keywords:** avian conservation, breeding success, *Corvus monedula*, ecological trap, food supplementation, urbanization

## Abstract

Urban areas expand worldwide, transforming landscapes and creating new challenging habitats. Some bird species, mainly omnivorous feeding on human waste and cavity nesters, commonly breed in these habitats and are, therefore, regarded as urban‐adapted. Although urban areas may provide new nesting sites and abundant human waste, the low breeding success found in some of these species suggests that the poor protein content in human waste might limit breeding parameters. We investigated whether the breeding success of a cavity nester and omnivorous species commonly breeding in urban areas, the Western Jackdaw (*Corvus monedula*), depended on the availability of good‐quality non‐urban food. We approached the objective by combining a literature review and experiments in the field. With the literature review, we compared jackdaw populations in different habitats across Europe and found that clutch size and number of fledglings per pair decreased with distance to non‐urban foraging grounds, even after controlling for the effect of colony size, latitude, and climate. In two experiments, we tested whether the breeding success of urban pairs could be increased by supplementing high‐quality food, first only during egg formation and second also until chick fledging. Food supplementation during egg formation led to larger eggs and higher hatching success than in urban control nests, but this did not result in higher chick survival. However, when food supplementation was prolonged until fledging in the second experiment, we observed a significant increase of nestling survival. These findings highlight that research and management actions should not only focus on species displaced by urbanization, but also on “urban‐adapted” species, as they might be suffering from a mismatch between availability of nesting sites in buildings and adequate non‐urban food resources. In these cases, nest sites should be provided in or close to adequate food resources.

## 1 Introduction

Urban areas develop worldwide at the expense of natural habitats and farmlands. During urbanization, much of the vegetation is replaced by impervious surfaces, such as buildings and roads. The structure of remnant vegetation is altered with substantial decreases in the shrub layer. Moreover, native species are often replaced by exotic ones. All this, together with the frequent use of pesticides in gardens and urban parks, leads to a decrease in biodiversity and, in particular, arthropods (Blair & Launer, 1997; Hengstum, Hooftman, Oostermeijer, & Tienderen, 2014; McKinney, 2002), leading to a lack of high‐quality food for birds in cities. On the other hand, buildings provide cavities, crevices, and elevated platforms, which may serve as roosting or breeding sites for some species.

Only species able to subsist on these reduced natural food resources and/or human waste, and to find suitable nesting sites will be able to occupy urban areas. Indeed, human waste has a too low protein content to replace natural food resources (Heiss, Clark, & McGowan, 2009), especially for chick rearing. Within birds, species subsisting in human settlements are mainly gregarious, monogamous, sedentary, and omnivorous, thus able to feed on anthropogenic food (Kark, Iwaniuk, Schalimtzek, & Banker, 2007). Urban areas can also be attractive because they provide the only breeding sites in certain landscapes. Therefore, many urban bird species are cavity breeders (Jokimäki, 1999), while ground or shrub nesters are scarce given the reduced understory vegetation (Rousseau, Savard, & Titman, 2015) and the threat imposed by domestic animals, such as cats (Loss, Marra, & Will, 2015).

However, even for species seemingly adapted to urban environments, reproductive output may be low in urban settlements compared with rural or natural habitats (Chamberlain et al., 2009). One of the main reasons put forward to explain this lower breeding success is the poor quality of food in urban settlements (Heiss et al., 2009; Sumasgutner, Nemeth, Tebb, Krenn, & Gamauf, 2014). Abundant human food waste and bird feeders in urban areas may provide resources for birds, but this food mainly consists of carbohydrates and fat. Thus, protein content of this diet may be too low for insectivorous species or chicks during growth (Heiss et al., 2009; Seress & Liker, 2015).

**Figure 1:**
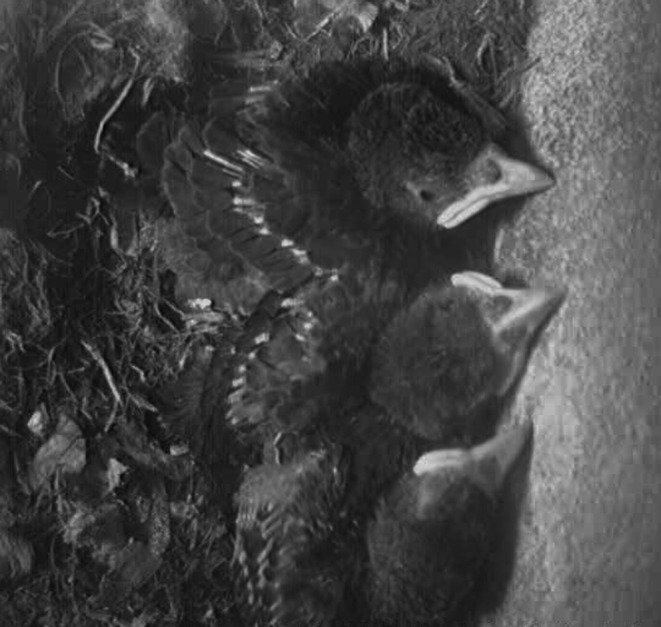
Nestlings of Western Jackdaw (*Corvus modedula*) about 17 days old

In this study, we used the Western Jackdaw (*Corvus monedula*; Figure 1) as a model to examine whether the lack of high‐quality food in cities is limiting breeding success even for species believed to thrive in urbanized habitats. The Western Jackdaw is omnivorous, monogamous, sedentary, colonial year‐round, and a secondary cavity nester (Dwenger, 1989; Röell, 1978). Therefore, a priori jackdaws may be expected to adapt to urban environments exploiting anthropogenic food and nesting in cavities in buildings. Indeed, populations of this species are found in both urban and agricultural or natural habitats (Dwenger, 1989), which makes jackdaws an excellent model for the study of the effects of urbanization.

To achieve our objective, we did a literature review and experiments in the field. In the review, we compared reproductive output across jackdaw populations varying in their accessibility (distance) to non‐urban foraging resources, while simultaneously accounting for other variables that might be important in driving breeding success, such as colony size. We predicted that clutch size and number of fledglings per pair would decrease with distance to non‐urban foraging grounds. With two experiments, we investigated whether breeding success in an urban colony was limited by available resources of good quality for birds, and therefore, whether it could be improved by providing supplementary food. In the first experiment, we food‐supplemented urban pairs before and during egg laying to test whether urban resources limit egg production and in turn breeding success. We hypothesized that food supplementation during egg formation would result in larger eggs and clutches and therefore in higher hatching success and chick survival (Knight, 1988), leading to reproductive success similar to jackdaws breeding in a nearby agricultural area. As this prediction proved only partly true, we added a second experiment in which we food‐supplemented urban pairs from pre‐laying until fledging of the young. Earlier observations in our colony have demonstrated a substantial starvation mortality of chicks in the nest (Strebel, 1991). We hypothesized that food resources available in urban areas are limiting chick growth, and thus, extending food supplementation until the end of the nestling period would be necessary to increase breeding success.

## 2 Dependence of Reproduction on Non‐urban Foraging Grounds: A Review

### 2.1 Literature search and meta‐analysis

We collected available information on clutch size and number of fledglings for 53 sites across Europe (Table S1). Data were obtained from scientific articles, books, dissertations, reports, and personal communications. In these, the number of fledglings is given as the number of nestlings about to fledge per breeding pair having initiated reproduction. We only used data from wild populations under natural conditions (no experiments, no culling). From the same or related sources, we gathered information on breeding site habitat, colony size (number of breeding pairs), and latitude because these variables have previously been related to breeding success in jackdaws (Dwenger, 1989; Kamiński, 1989; Soler & Soler, 1992). Weather data for each site and year were obtained from nearby meteorological stations through the KMNI climate explorer (http://climexp.knmi.nl/).

We applied two Gaussian mixed models to investigate the impact of breeding habitat on reproductive performance, while simultaneously testing for the effect of colony size, latitude, and weather variables. The dependent variables were clutch size and number of fledglings, both expressed as average per site and year. The effect of *breeding habitat* was tested as an explanatory factor with five categories: agricultural fields (*Agricultural*), forested area (*Wood*), and urban (nesting in buildings within urban settlements) at close (<100 m; *UrbanC*), intermediate (100–500 m; *UrbanI*), or far distances (>500 m; *UrbanF*) from non‐urban foraging areas (mainly agricultural fields). The cutoff point at 100 m was used to describe colonies with “direct” access to non‐urban forage, while the one at 500 m reflects own observations of GPS‐logged jackdaws (see below). The independent variable *colony size* was log‐transformed, because reproduction likely does not decrease linearly with colony size. We also controlled for the linear and quadratic effects of *latitude* and rainfall (site‐ and year‐specific; in mm). *April rainfall* was used in the clutch size model, while for the analysis of number of fledglings we used *May rainfall* and *June rainfall*. Temperature was not included due to its high correlation with latitude. Finally, we used three random factors to account for the autocorrelation among data within the same study (*source*) and population (*site*), as well as for inter‐annual variations in breeding performance (*year*; Table S2).

Analyses were performed in the program R (version 2.15.1; R Development Core Team 2015) with the function *lmer* from package lme4 (Bates, Maechler, Bolker, & Walker, 2015). Effects were assessed using the Bayesian framework. We simulated a random sample (*N* = 5,000) from the joint posterior distribution of the model parameters using the function *sim* from package arm (Gelman et al., 2010). From this sample, we used the 2.5% and 97.5% quantiles as lower and upper limit of the 95% credible interval (CrI), and an effect was considered significant when the 95% CrI did not contain zero. For the case of categorical variables (e.g., habitat type), we additionally calculated the posterior probability of the hypothesis that the mean number of eggs or fledglings in one habitat type is different from that in the other habitat for all pair‐wise combinations of habitat categories. The higher this probability is, the stronger is the difference between categories. We considered that the means differed significantly when the probabilities were larger than 0.975, which would be analogous to a two‐tailed *p*‐value of .05 (Schmidt et al., 2013; Wilkes et al., 2013).

### 2.2 Results and discussion

**Figure 2:**
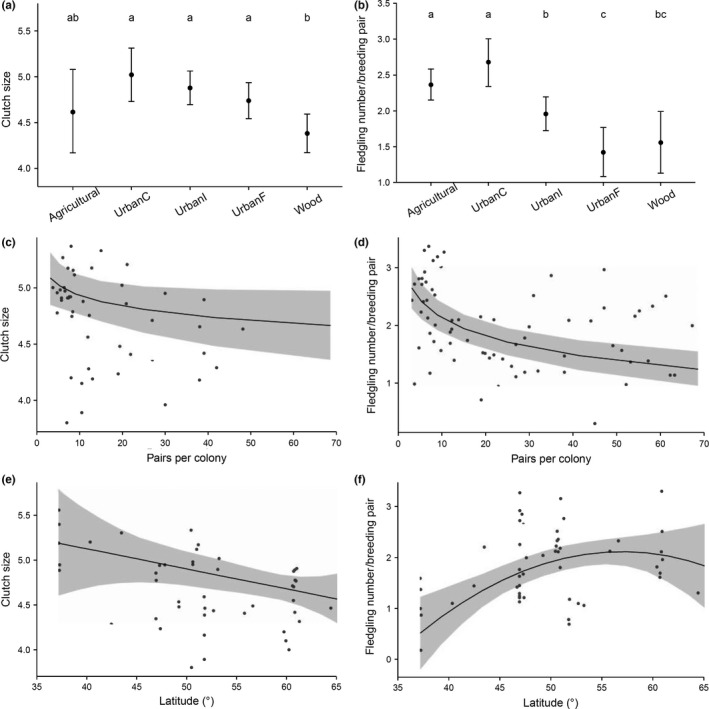
Clutch size (left column) and number of fledglings (right column) (mean fitted values ± 95% CrI) according to breeding habitat (a, b), colony size (c, d), and latitude (e, f). Shaded areas are the 95% CrI given by the model (Table S2) for the estimated trend line. Dots are raw data. *Agricultural* = breeding in agricultural areas (*N* = 80), *Urban *= breeding in urban settlements with non‐urban foraging areas within 100 m (*UrbanC*;* N* = 36), within 100–500 m (*UrbanI*;* N* = 80), or further than 500 m (*UrbanF*;* N* = 39), and *Wood *= breeding in woodlands (*N* = 41). In the top panels, predicted means are significantly different from each other (i.e., they differ with a posterior probability larger than 0.975) when they do not share the same letters

Clutch size in urban breeding sites tended to decrease, although not significantly, with the distance to non‐urban foraging areas (from close to far, i.e., *UrbanC *> *UrbanI* > *UrbanF*), while clutch size in agricultural areas showed a large range of values (Table S2; Figure 2a). The number of fledglings was similar in agricultural breeding sites and urban sites close to non‐urban (mainly agricultural) foraging grounds (*UrbanC*) and decreased strongly with increasing distance to these foraging grounds (i.e., *UrbanC *> *UrbanI* > *UrbanF*; Table S2; Figure 2b). These findings are in agreement with studies on other bird species, including corvids, comparing urban to non‐urban populations, which generally show lower productivity in urban areas (Chamberlain et al., 2009; Heiss et al., 2009; Richner, 1989; Sumasgutner et al., 2014). The negative link between breeding parameters and distance to non‐urban foraging areas indicates that the lack of natural food in urban areas may be driving the low breeding success, as hypothesized by Chamberlain et al. (2009). Interestingly, clutch size and number of fledglings were lowest for jackdaws breeding in woodlands. Reasons may be that part of these pairs bred in natural tree holes, which generally have narrower bottoms than nest boxes and cavities in buildings (Löhrl, 1973), thus, entail smaller clutches and that predation in woods may be higher than in urban areas, leading to smaller clutch sizes and fledgling numbers (Johnsson, 1994; Yom‐Tov, 1974). In addition, forest‐breeding jackdaws may have long foraging distances, as they do not usually forage in wooded areas.

Clutch size and number of fledglings decreased significantly with *colony size* (Table S2; Figure 2c, d). This might result from an increase in competition for nests, and thus, agonistic interactions (Antikainen, 1978; Röell, 1978), and from increased competition for food resources (Henderson & Hart, 1995).

Latitude had a negative effect on clutch size (Table S2; Figure 2e), as already observed by Soler and Soler (1992), who suggested that jackdaws at northern latitudes favoured larger egg sizes over larger clutch sizes. The number of fledglings, on the other hand, increased with latitude in a curvilinear way (Table S2; Figure 2f), which could be explained by the limiting effects of dry summers in the south and cold springs in the north. Neither clutch size nor number of fledglings was significantly associated with rainfall.

## 3 Food Supplementation Experiments

### 3.1 Description of the study colony

For both experiments, we used a long‐established colony of about 26 reproductive pairs (average 1989–2015) breeding in nest boxes in a castle in the middle of Murten (46°55′41″N 7°06′55″E), a town of 6,550 inhabitants in the Swiss lowlands (elevation 500 m, mean annual temperature 9°C, precipitation 900 mm). The surrounding countryside consists of agricultural land and scattered woods (nearest agricultural field at about 500 m from the breeding site).

**Figure 3:**
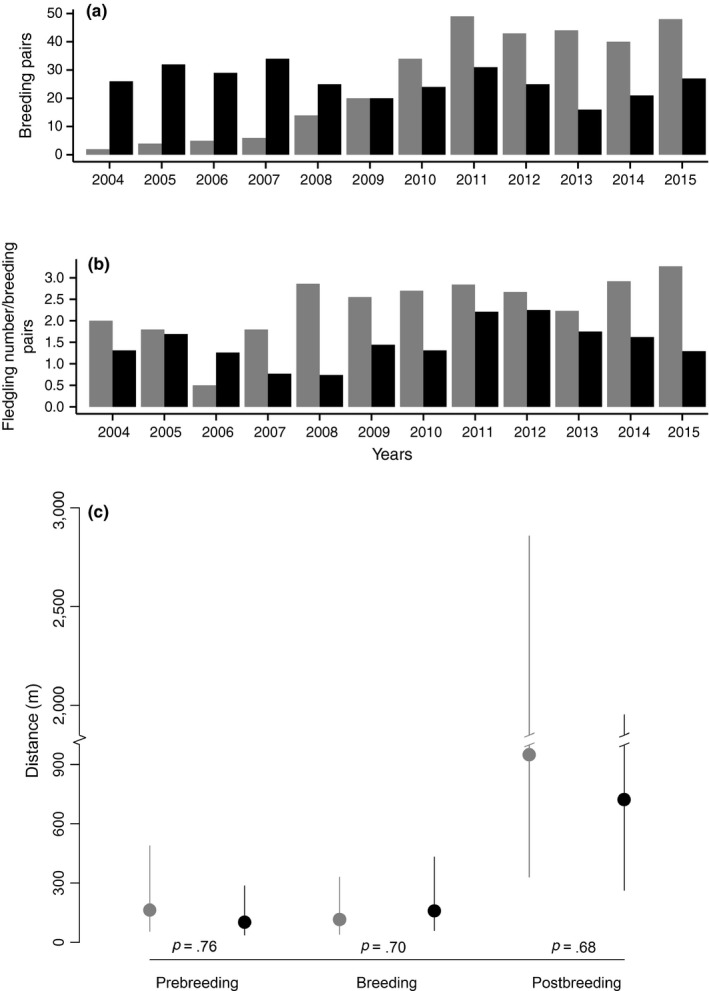
Number of breeding pairs (a) and fledglings per breeding pair (b) in the urban (black; Murten) and agricultural (gray; Kerzers/Galmiz) sites between 2004 and 2015. From 2004 onwards, four to eight nest boxes were put on eight electrical pylons in the agricultural area and the number of breeding pairs increased from 0 to 54. (c) Mean distance from locations of jackdaws equipped with GPS loggers to their nesting place in Murten (black) and Kerzers/Galmiz (grey) during the pre‐breeding period (30 days before egg laying; *N* = 265 fixes of two birds), breeding period (55 days from egg laying until fledging; *N* = 1,285 fixes of eight birds), and post‐breeding period (30 days after fledging; *N* = 1,061 fixes of eight birds). Dots are the mean fitted values ±95% CrI according to the model in Table S5. *P* are the posterior probabilities that the means between Murten and Kerzers/Galmiz are different within each period. The higher this probability is, the stronger is the difference

The reproductive success of this urban colony, determined by checking all nest boxes at least once during the breeding peak, is particularly low when compared with jackdaws breeding in a nearby agricultural area (5.5 km away; thus, under the same weather conditions) between the villages of Galmiz (46°56′59″N, 7°09′25″E) and Kerzers (46°58′30″N, 7°11′44″E). The urban colony produced on average 1.47 fledglings per reproductive pair, while pairs in the agricultural area produced around 2.35 fledglings (Figure 3). Strebel (1991) showed that chick mortality in Murten was high and explained this by his observation of parents feeding chicks partly with human waste.

Adult jackdaws equipped with GPS loggers in Murten and Kerzers/Galmiz (see supporting information S3 for details) showed that during the pre‐breeding (30 days before egg laying) and breeding (incubation and chick rearing) periods, jackdaws stayed near the nesting sites (average distance from nest about 160 m, upper 95% CrI at about 500 m; Figure 3c). Thus, birds from Murten were mainly restricted to urban resources and only partially visited agricultural areas further away, while jackdaws breeding in Kerzers/Galmiz had continuous access to agricultural food resources (Figure S4). During the post‐breeding period (30 days after chick fledging), however, jackdaws used areas much farther from the nesting sites, leading to an overlap of the range used by birds from the urban and agricultural sites (Figure S4; Table S5).

### 3.2 Experimental design

We used 15 (2014; first experiment) and 19 (2015; second experiment) all similar nest boxes placed in the same main tower of the Murten castle. In the first experiment, we food‐supplemented half of the nest boxes during egg formation (from about 20 days prior to egg laying until the last egg of the given nest box was laid). Daily, we supplied every other nest box (*U_Fed*;* n* = 7) with about 60 g of scrambled eggs (as Soler & Soler, 1996) in a metal cup inside the nest box (Figure S6). The remaining nest boxes (*U_Con*;* n* = 8) were subjected to the same protocol but without adding food.

In 2015, we repeated the same procedure; however, to avoid nest box‐specific biases, we inverted the treatment, so that fed nest boxes in 2014 became controls in 2015, and vice versa (in 2015 *U_Fed* = 10 nest boxes and *U_Con* = 9). We prolonged the food supplementation of *U_Fed* nests until chicks fledged. During incubation, we continued with 60 g of eggs daily, while during chick rearing, we changed to supply insect maggots (mainly mealworms): 5–6 g per chick per day until chicks were 2 weeks old and 8–9 g per chick and day afterward.

Infrared (IR) cameras inside the nest boxes (see below; Figure S6) confirmed that jackdaws accepted the supplemented food within a few days during the pre‐laying period and that parents were feeding the maggots to the chicks. The 60 g of scrambled eggs, available to both parents, represent about 90% of the daily food ration of one adult jackdaw (not taking into account egg production; Nagy, 2001). The maggots represent about 50% of the daily food requirement of young chicks and 7–10% of the daily food requirement of older chicks (Kamiński, 1986). Therefore, we did not replace, but only supplemented the normal chick diet.

In both experiments, we recorded laying date, clutch size, egg mass at laying (±0.1 g), egg length and width (±0.1 mm), hatching date and success, and chick weight and survival until day 27. We used egg length and width to calculate egg volume (cm^3^) according to Hoyt (1979; volume = 0.51 × length × (width)^2^). Four *U_Fed* and four *U_Con* nest boxes, equally distributed around the tower, were equipped with IR cameras in both years to verify the use of supplemented food, and to examine whether added food altered the frequency at which adults fed their chicks.

In 2014, we had the opportunity to compare the performance of the urban colony with that of birds breeding in the agricultural area. We installed IR cameras in eight nest boxes on two electrical pylons between Kerzers and Galmiz to record laying date, clutch size, hatching date and success, chick survival, and chick‐feeding frequency. These nest boxes could only be accessed by climbing the pylons; thus, direct nest checks (*A_Con*;* n* = 9) were restricted to only one visit during egg laying, in which we could measure egg size but not weight at laying, one visit at hatching, and one at 17 days. All work was carried out while minimizing disturbance to birds. Permissions for catching and marking birds and for the feeding experiments were given by the Service de la Sécurité Alimentaire et des Affaires Vétérinaires of the Canton of Fribourg and the Federal Office for the Environment.

### 3.3 Statistical analyses

We performed two sets of analyses. First, to investigate the effect of food supplementation during egg formation, we used the data from the first experiment in Murten (*U_Fed* and *U_Con* 2014) and the additional control group in the agricultural area (Kerzers/Galmiz; *A_Con*). Second, to test whether urban resources limit chick rearing, we compared reproductive parameters of urban nests fed from pre‐laying until chick fledging in the second experiment (*U_Fed* 2015) with control urban nests of the same year (*U_Con* 2015), as well as with fed and control urban nests of 2014, when food was provided only during egg formation (*U_Fed* and *U_Con* 2014).

The first set of analyses (Experiment 1; 2014) consisted of 11 linear or linear‐mixed models (Table 1) in which we tested the effect of *food treatment* (*U_Fed, U_Con, A_Con*): first on clutch size, egg volume, incubation duration as well as the body weight of hatchlings and 17‐day‐old chicks, which best followed a normal distribution. Then, we examined the treatment effect on hatching success and nestling survival until day 5, 10, 17, and 27, with a binomial error structure, and finally on the feeding frequency, which best fitted a poisson distribution. Note that the effect of *food treatment* on start of laying was not included in these models because with our experimental design we did not cause an earlier laying date (mean Julian laying date *U_Fed 2014* was 109.9 days and *U_Fed 2015* was 114.3 days vs. *U_Con 2014* with 110.1 days and *U_Con 2015* with 114.0 days; *F*‐test *p*‐value_2014_ = .8; *p*‐value_2015_ = .7). The incubation duration is the number of days between the laying of the second egg and the hatching of the first egg. Hatching success represents the proportion of eggs that hatched, while chick survival is the proportion of hatched chicks reaching day 5, 10, 17, and 27, respectively. Feeding frequency is the number of parental feeding visits recorded from 6 to 8 a.m. and from 6 to 8 p.m., when bird feeding has been shown to be maximal (Henderson & Hart, 1993). Nests that failed during incubation due to causes independent of the treatments (e.g., predation) were omitted from chick survival analyses.

**Table 1:** Models implemented in the first set of analyses comparing urban nests fed during egg formation with urban control nests and agricultural control nests in 2014

Models	*N*	Distribution	Response variables	Explanatory variables	Random factors
Model 1	24	Normal	Clutch size	Food treatment 2014 + Laying date + Mean temperature	‐
Model 2	109	Normal	Egg volume	Food treatment 2014 + Laying date + Clutchsize + Mean temperature + Number of rainy day + (Number of rainy day)^2^	brood_ID
Model 3	22	Lognormal	Incubation duration	Food treatment 2014 + Laying date + Clutchsize + Mean temperature	‐
Model 4	84	Normal	Hatchling weight	Food treatment 2014 + Hatching date + Mean temperature + Number of rainy day + (Number of rainy day)^2^	brood_ID
Model 5	44	Normal	Chick weight (17 days)	Food treatment 2014 + Hatching date + Mean temperature + Number of rainy day + (Number of rainy day)^2^	brood_ID
Model 6	24	Binomial	Hatching success	Food treatment 2014 + Hatching date + Mean temperature	‐
Model 7–10	24	Binomial	Survival at 5, 10, 17 or 27 days	Food treatment 2014 + Hatching date + Mean temperature	‐
Model 11	689	Poisson	Feeding frequency	Food treatment 2014 + Chick age + (Chick age)^2^ + log(Brood size) + Daily mean temperature + Daily rainfall	brood_ID + date

NB, Weather data are specific for each period.

As previous studies have shown a link between weather conditions and reproductive parameters in jackdaws (Kamiński, 1989), we accounted for these potential effects by including *mean temperature* (°C) and, where sample size permitted, also the *number of rainy days* (at least 1 mm of rainfall) in the models. We used *number of rainy days* instead of the amount of precipitation to avoid correlation with *mean temperature* and to obtain a measure of the temporal extent of rainfall. We also included the quadratic term of *number of rainy days* as more precipitation may lead to more food availability (vegetation and insects), while too much rain may result in lower food resources and reduced foraging activity (Hogstedt, 1981). Weather records were obtained from the Federal Office of Meteorology and Climatology MeteoSwiss (http://www.meteoswiss.admin.ch/home.html) for locations near the study site. Calculations were specific for each nest and period. For clutch size and egg parameters, we used weather data from 1 week before the start of laying until the end of laying, for the incubation duration, hatchlings weight, and hatching success, we used records of the whole incubation period, and for chick weight and survival models, we used weather data from hatching date until the model‐specific chick age (5, 10, 17, or 27 days). For feeding frequency, we used the daily *mean temperature* (°C) and *rainfall* (in mm) of the days when feeding frequency was recorded.

Apart from *food treatment* and weather conditions, we also accounted for the effect of additional variables (see Table 1 for specific explanatory variables being used in each model): *Laying date* (in Julian days), *clutch size*,* hatching date*,* brood size* (number of chicks per nest and day, log‐transformed to account for the fact that parental effort is limited and reaches an asymptote at larger brood sizes), *chick age* (average age per nest; linear and quadratic effect, given that chick food requirements, and thus feeding frequency, tend to increase with chick growth, but decrease again before fledging; Harris, 1978). Two random factors were used to account for autocorrelation among eggs or chicks within a nest (*brood_ID*) and for random variability among specific dates (*date*) (see Table 1 for details).

The second set of analyses consisted of repeating the same models as for the first set, but adapting them to the combined data from both experiments (see Table 2 for specific explanatory variables being used in each model). That is, we included *year* as fixed effect (2014 vs. 2015) and its interaction with *food treatment* (*U_Fed* vs. *U_Con*) in order to compare the effect of supplementing food only during egg formation (2014) versus until chick fledging (2015). As the nest boxes followed were the same in both years, we included the nest box (*nestbox_ID*) as further random factor. Given that for this set of analyses we used more detailed data from Murten, we were able to explore the effect of *food treatment* on egg weight at laying and on chick weight at 0, 5, 10, 17, and 27 days (Table 2).

**Table 2:** Models implemented in the second set of analyses comparing fed urban nests with control urban nests in the frame of two different experiments (1: supplementary feeding only during egg formation in 2014; 2: supplementary feeding until chick fledging in 2015)

Models	*N*	Distribution	Response variables	Explanatory variables	Random factors
Model 12	34	Normal	Clutch size	Food treatment (urban; both years pooled) * Years + Laying date + Mean temperature	nestbox_ID
Model 13	161	Normal	Egg volume	Food treatment (urban; both years pooled) * Years + Laying date + Clutch size + Mean temperature + Number of rainy day + (Number of rainy day)^2^	nestbox_ID + brood_ID
Model 14	161	Normal	Egg weight	Food treatment (urban; both years pooled) * Years + Laying date + Clutch size + Mean temperature + Number of rainy day + (Number of rainy day)^2^	nestbox_ID + brood_ID
Model 15	34	Lognormal	Incubation duration	Food treatment (urban; both years pooled) * Years + Laying date + Clutch size + Mean temperature	nestbox_ID
Model 16	99	Normal	Hatchling weight	Food treatment (urban; both years pooled) * Years + Hatching date + Mean temperature + Number of rainy day + (Number of rainy day)^2^	nestbox_ID + brood_ID
Model 17	62	Normal	Chick weight (5 days)	Food treatment (urban; both years pooled) * Years + Hatching date + Mean temperature + Number of rainy day + (Number of rainy day)^2^	nestbox_ID + brood_ID
Model 18	54	Normal	Chick weight (10 days)	Food treatment (urban; both years pooled) * Years + Hatching date + Mean temperature + Number of rainy day + (Number of rainy day)^2^	nestbox_ID + brood_ID
Model 19	47	Normal	Chick weight (17 days)	Food treatment (urban; both years pooled) * Years + Hatching date + Mean temperature + Number of rainy day + (Number of rainy day)^2^	nestbox_ID + brood_ID
Model 20	41	Normal	Chick weight (27 days)	Food treatment (urban; both years pooled) * Years + Hatching date + Mean temperature + Number of rainy day + (Number of rainy day)^2^	nestbox_ID + brood_ID
Model 21	34	Binomial	Hatching success	Food treatment (urban; both years pooled) * Years + Hatching date + Mean temperature	nestbox_ID
Model 22–25	34	Binomial	Survival at 5, 10, 17 or 27 days	Food treatment (urban; both years pooled) * Years + Hatching date + Mean temperature	nestbox_ID
Model 26	269	Poisson	Feeding frequency	Food treatment (urban; both years pooled) * Years + Chick age + (Chick age)^2^ + log(Brood size) + Daily mean temperature + Daily rainfall	nestbox_ID + brood_ID + date

NB, Weather data are specific for each period.

All analyses of this section were performed using the same program and framework as in the literature review, but we varied the function used as well as the simulated random sample (*N* = 5,000) (*lm, lmer, or glmer*) according to the model (Tables 1, 2).

### 3.4 Results of food supplementation experiments

**Figure 4:**
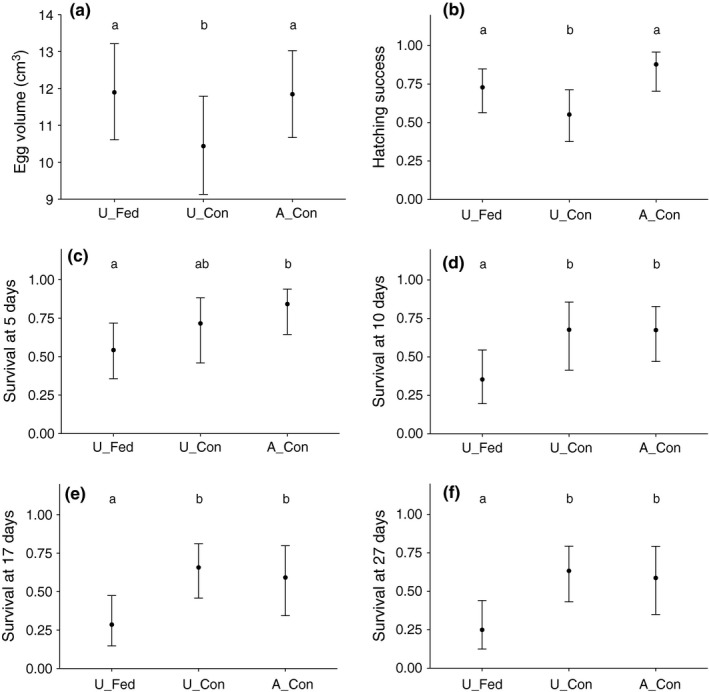
Effect of food supplementation during egg formation (experiment 1; 2014) on egg volume (a), hatching success (b), and chick survival () until day 5 (c), 10 (d), 17 (e), and 27 (f). Dots are the mean fitted values ± 95% CrI according to models and sample sizes in Table 1. *U_Fed* = food‐supplemented urban nests; *U_Con* = urban control nests; *A_Con* = agricultural control nests. Predicted means are significantly different from each other (i.e., they differ with a posterior probability larger than 0.975) when they do not share the same letters

In the first set of analyses, considering food supplementation during egg formation in 2014, food supply did not significantly affect clutch size (mean ± *SD U_Fed* = 4.88 ± 1.13; *U_Con* = 4.86 ± 0.96; *A_Con* = 5.38 ± 0.74) or incubation duration (mean ± *SD U_Fed* = 18.38 ± 0.52; *U_Con *= 19.14 ± 1.07; *A_Con* = 19 ± 0.01). However, food supplementation resulted in significantly larger eggs in *U_Fed*, compared with the unfed control group in Murten (*U_Con*), which reached the volume of those in the agricultural area (*A_Con*; Figure 4a; Table S7). We did not find a significant relationship between egg parameters (clutch size, incubation duration, egg volume) and any further explanatory variable tested (Tables 1 & S7).

Nestling weight was not influenced by any of the model parameters (Table S8). The hatching success in nests food‐supplemented during egg formation was higher than in urban control nests and reached values similar to those of the agricultural site (Figure 4b; Table S9). Chick survival until day 5 (proportion of hatchlings reaching day 5) was almost similar in the three groups (Figure 4c; Table S9). However, chick survival until day 10, 17, and 27 in the food‐supplemented group was lower than in both control groups (i.e., Murten and the agricultural area; Figure 4d–f; Table S9), indicating that at day 5 chick mortality in nests food‐supplemented during pre‐laying and laying was higher than in control nests. Independent of treatment, *hatching date* had a significant negative effect on chick survival. We did not find a significant effect of weather conditions on hatching success or chick survival (Table S9). Feeding rates were not affected by food supplementation during egg formation or by weather conditions, but increased with brood size in an asymptotic way and showed a quadratic response to nestling age (Table S9).

**Figure 5:**
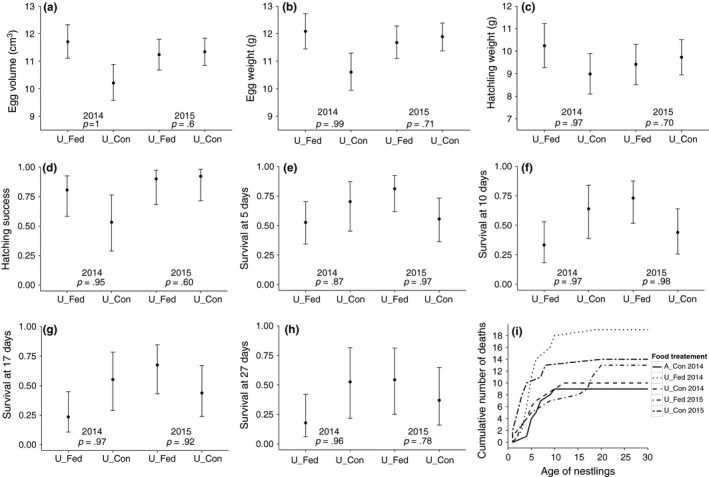
Results of the second set of analyses comparing fed urban nests with control urban nests in the course of two experiments (1: supplementary feeding only during egg formation in 2014; 2: supplementary feeding until chick fledging in 2015). Effect of supplementary feeding on egg volume (a), egg weight (b), hatchling weight (c), hatching success (d), and chick survival until day 5 (e), 10 (f), 17 (g), and 27 (h). (i) Cumulative number of dead nestlings according to age for the experimental groups. Dots are the mean fitted values ± 95% CrI according to models and sample sizes in Table 2. *U_Fed* = urban nests that were food supplemented until the end of egg laying (*N*_2014_ = 8) or chick fledging (*N*_2015_ = 9). *U_Con* = urban control pairs (*N*_2014_ = 7; *N*_2015_ = 10). *A_Con* = agricultural control nests (*N*_2014_ = 9). *P* are the posterior probabilities that the means differ between the fed and control groups within each year. The higher this probability is, the stronger is the difference

In the second set of analyses, we combined experiment 1 (food supplementation during egg formation) and experiment 2 (food supplementation during egg formation, incubation, and chick rearing). As in the first set of analyses, clutch size was not significantly affected by food supplementation (mean ± *SD U_Fed 2014 *=* *4.88 ± 1.13; *U_Con 2014 *=* *4.86 ± 0.96; *U_Fed 2015 *=* *4.89 ± 0.6; *U_Con 2015 *=* *4.41 ± 0.84) nor was it significantly influenced by year, the interaction of year with food treatment (experiment 1 vs. experiment 2), laying date, or weather conditions (Table S10). In the models for egg volume and weight, the interaction between year and food treatment was significant. That is, while in 2014 the eggs of fed nests were significantly larger and heavier than those of unfed nests, in 2015 this difference was not apparent, as both groups (*U_Fed* and *U_Con 2015*) had large, heavy eggs (Figure 5a, b; Table S10). Across treatments, egg parameters decreased with laying date. Also, egg volume increased with temperature, while precipitation did not show any substantial effect (Table S10). The duration of incubation was significantly shorter (i.e., by around 1 day) in food‐supplemented nests (Table S10). Similarly to the first experiment, we did not find a significant influence of clutch size, laying date, or weather on incubation duration.

Hatchling weight was correlated with food treatment in 2014 (Figure 5c; Table S11), while this effect disappeared with time during the rearing period. The difference in hatching success found in 2014 between fed and control nests was not apparent in 2015, as in 2015 it was high for both fed and control nests (Figure 5d; Table S12), which agrees with the models for egg volume and weight. Regarding chick survival, the significant interaction between *year* and *food treatment* showed that contrarily to the first experiment (2014), when nests were fed only until the end of laying, food supplementation during chick rearing (*U_fed* in 2015) increased chick survival until day 17, when compared with control nests (*U_Con* in 2015). However, this trend disappeared in the model of chick survival until 27 days (Figure 5e–h; Table S12), which seems to be caused by higher chick mortality after day 17 in nests being food‐supplemented during chick rearing (Figure 5i). The mortality in all other treatment groups and years generally occurred at day 10. As in the first set of analyses, weather conditions did not have a significant effect on hatching success or chick survival. However, *hatching date* did not have an effect on chick survival. Although there are significant differences in feeding frequency between years, they appear to be independent of *food treatment* (Table S12). In this analysis, feeding rates increased again with brood size, but were not significantly dependent on chick age or weather conditions (Table S12).

### 3.5 Discussion

We found an effect of supplementary feeding on some breeding parameters suggesting that the lack of high‐quality food resources in urban environments may be limiting breeding success, even for an omnivorous species able to feed on human waste. We did not find a significant effect of the supplementary feeding on clutch size, which can be explained by jackdaws following a brood‐reduction strategy and thus adjusting brood size rather than clutch size (Soler & Soler, 1996). However, we observed that food supplementation during egg formation entailed larger eggs in fed urban nests in 2014, which, in turn, led to higher hatching success, reaching values found in the agricultural area. This may be explained by the fact that the lower content of protein in the diet of urban jackdaws, which feed, partly, on human waste, is limiting the size of the eggs and hence their hatching success. This agrees with previous studies reporting that birds supplemented with protein‐rich food laid larger eggs than birds supplemented with low‐protein diet (Nager, 2006; Soler & Soler, 1996).

Despite the larger eggs and higher hatching success, the overall breeding success was not improved by this first experiment. This was due to chick survival being lower in the fed than in the control group, especially after day 5 when the reserves of the yolk sac are used up (Noy & Sklan, 2001). Food supplementation during egg formation might have led pairs to anticipate optimal breeding conditions (Seress & Liker, 2015); however, when food supplementation was stopped, parents may have been unable to cope with rearing the larger numbers of chicks hatched. Parental chick‐feeding frequency was not altered by the treatment, confirming that urban fed parents might not have been able to compensate for the extra number of nestlings, thus leading to higher nestling mortality than in urban and agricultural control nests.

With the second experiment, we corroborated that shortage of good‐quality food in urban areas is limiting jackdaw reproductive success through decreased nestling survival (as suggested by Chamberlain et al., 2009 and Heiss et al., 2009). Prolonging food supplementation until chick fledging significantly improved nestling survival compared with urban control nests, as opposed to the situation found in the first experiment. Unfortunately, this positive effect of supplementary feeding disappeared toward the end of the nesting period as shown in the temporal pattern of mortality. In nests fed until fledging, mortality occurred mainly when chicks reached adult size (17–20 days), while in control nests and nests fed only during egg formation chick mortality occurred mainly in the first 10 days, which is the usual pattern (Heeb, 1994; Kamiński, 1989). This could result from a shortcoming in our experimental design, as the amount of food supplemented might not have been enough to sustain chicks when they became larger. This is supported first by our observation that parental feeding frequency did not decrease in fed nests, while sufficient food supplementation normally reduces parental feeding frequency (Bolton, 1995) and second by the fact that fed chicks of 17 days old were not heavier than in the other groups.

Surprisingly, in this second experiment, we did not observe an increase in egg dimensions or hatching success with food supplementation. This could be due to environmental conditions. Temperature during the laying period in 2015 was on average two degrees higher than in 2014; thus, natural food availability might have been better in 2015. This could have allowed also non‐fed pairs to produce larger eggs, thus alleviating the effect of food supplementation on egg quality (Boutin, 1990; Schoech et al., 2007). This agrees with the positive link we found between temperature and egg mass and volume. Food supplementation, on the other hand, seemed to shorten the duration of incubation suggesting that food might have allowed females to stay longer incubating instead of foraging.

## 4 Conclusion

Both the literature review and the experiments demonstrated that food resources in urban areas were limiting reproduction for an omnivorous species able to use human waste. Also other omnivorous species (corvids, house sparrow), commonly breeding in cities, appear to suffer from food limitations in human settlements (Heiss et al., 2009; Peach et al. 2015; Richner, 1989, 1992). They have to either travel long distances to reach high‐quality food outside the urban area or rely on low‐quality human waste, which contains two to three times less protein than invertebrates (Heiss et al., 2009) and also less calcium (Pierotti & Annett, 1990). Both options may then lead to poor‐quality eggs, high nestling mortality, and reduced breeding success. Nevertheless, jackdaws seem to be attracted to urban areas by the availability of nesting sites. Especially for cavity nesters, natural breeding sites have decreased dramatically due, partly, to the removal of old trees. Thus, some species might be forced to breed in buildings, creating a mismatch between adequate foraging grounds and breeding sites. Our study demonstrates this mismatch and indicates that species regularly breeding in urban settlements might be experiencing an ecological trap (sensu Battin, 2004).

Future studies should investigate demographic parameters other than breeding success (e.g., adult survival or emigration/immigration) to fully understand the consequences of living in cities and whether urban areas are indeed ecological traps for some species. However, this work already emphasizes that conservation measures should not only focus on species, which are displaced by urbanization (Brown & Graham, 2015). It is also important to pay attention to species commonly seen in human settlements as they might be suffering from reduced breeding success due to lack of non‐urban breeding sites. Conservation measures for such species seemingly thriving in urban areas should mitigate the mismatch between food and nest site availability. This could be performed, on the one hand, by “renaturalizing” urban green areas (e.g., by reducing exotic plant species or use of pesticides) to enhance biodiversity and hence natural high‐quality food, and on the other hand, by providing and/or preserving nest sites in non‐urban areas with adequate foraging areas (e.g., nest boxes, old trees, or buildings with cavities for hole nesters). Given the accelerating course of urbanization worldwide, understanding the consequences for population dynamics will be essential to allow the future coexistence of wildlife and urban areas.

## Conflict of Interest

None declared.
