# Supplementary material for: Happy to breed in the city? Urban food resources limit reproductive output in Western Jackdaws
Source: Ecol Evol. 2017 Feb 1;7(5):1363–74. doi: 10.1002/ece3.2733 (PMC5330913; doi:10.1002/ece3.2733)
Supplement: Supplementary file 1 [file ECE3-7-1363-s001.doc]

Supporting information

**Table S1: European jackdaw populations incorporated in the literature review.**

| **References** | **Latitude** | **Country** | **Years** |
| --- | --- | --- | --- |
| Antikainen 1978; Antikainen 1981 | 60.2512 | Finland | 1969, 1971-74 |
| Antikainen 1978; Antikainen 1981 | 60.4518 | Finland | 1966-1969 |
| Antikainen 1978; Antikainen 1981 | 60.4556 | Finland | 1966-1974 |
| Antikainen 1978; Antikainen 1981 | 60.4661 | Finland | 1966-1969 |
| Antikainen 1978; Antikainen 1981 | 60.5422 | Finland | 1969, 1971-74 |
| Antikainen 1978; Antikainen 1981 | 60.5616 | Finland | 1966-1969 |
| Antikainen 1978; Antikainen 1981 | 60.6785 | Finland | 1966-1974 |
| Antikainen 1978; Antikainen 1981 | 60.6821 | Finland | 1966-1969 |
| Antikainen 1978; Antikainen 1981 | 60.8091 | Finland | 1966-1969 |
| Antikainen 1978; Antikainen 1981 | 60.8742 | Finland | 1966-1969, 1971-1974 |
| Antikainen 1978; Antikainen 1981 | 61.1308 | Finland | 1966-1969 |
| Antikainen 1978; Antikainen 1981 | 63.1182 | Finland | 1966-74 |
| Antikainen 1978; Antikainen 1981 | 64.2930 | Finland | 1966-74 |
| Antikainen 1978; Antikainen 1981 | 64.8075 | Finland | 1966-74 |
| Arguello and Alvarez 1986 | 42.5190 | Spain | 1984-1985 |
| Biondo 1998; Strebel 1991; Koller unpublished; Meyrier unpublished; Tablado unpublished, Vogel unpublished | 46.9280 | Switzerland | 1989-1994, 2003-2015 |
| Chesney 1986 | 56.5188 | England | 1983-1984 |
| Domingez 1999 | 40.4180 | Spain | 1984-1985 |
| Dwenger 1989 | 50.9641 | Germany | 1978-1986 |
| Dwenger 1989 | 51.2700 | Germany | 1980-1981 |
| Dwenger 1989; Freistaadt Thuringen 2010 | 50.8750 | Germany | 1973-2004 |
| Ejgelis 1958 | 59.9400 | Russia | 1955 |
| Folk 1968 | 49.2053 | Czech Republic | 1959-1960 |
| Gibbons 1987 | 43.5182 | France | 1985 |
| Henderson and Hart 1993 | 52.6300 | England | 1987-1991 |
| Hogstedt 1977 | 55.6955 | Denmark | 1976 |
| Hund and Prinzinger 1981 | 47.8092 | Germany | 1974-1979 |
| Johnsson 1994 | 59.7497 | Sweden | 1986-1991 |
| Kaminski and Konarzewski 1984; Kaminski 1989 | 53.0496 | Poland | 1981-86 |
| Lockie 1955; Heeb 1994; Arnold & Griffiths 2003 | 51.7750 | England | 1950-54, 1986-1989 |
| Mayaud 1933 | 47.0909 | France | 1925 |
| Metz, Neitzsch & Schindler 1983 | 50.4903 | Germany | 1971-79 |
| Meyrier unpublished; Tablado unpublished, Vogel unpublished | 46.9572 | Switzerland | 2008-2014 |
| Pikula & Folk 1970 | 49.1884 | Czech Republic | 1961-1968 |
| Richford & Lawman1978 | 51.6983 | Wales | 1975-1976 |
| Richford & Lawman1978 | 51.7365 | Wales | 1975-1976 |
| Riggenbach 1979 | 46.6919 | Switzerland | 1961-1965 |
| Riggenbach 1979 | 47.1700 | Switzerland | 1972-1975 |
| Riggenbach 1979 | 47.3048 | Switzerland | 1952-1973 |
| Riggenbach 1979 | 47.5948 | Switzerland | 1964-1976 |
| Roell 1978 | 53.1780 | Belgium | 1971-75 |
| Schmidt 1988; Schmidt & Schmidt 1994 | 50.5800 | Germany | 1987-1993 |
| Schmidt 2004 | 50.6839 | Germany | 2002 |
| Schoof 1977 | 51.1190 | Germany | 1950 |
| Soler 1984; Soler 1990 | 37.3050 | Spain | 1979-1983 |
| Soler 1984; Soler 1990 | 37.3243 | Spain | 1981 |
| Soler 1984; Soler 1990 | 37.3500 | Spain | 1980-1982 |
| Soler 1984; Soler 1990 | 37.3681 | Spain | 1980 |
| Soler 1984; Soler 1990 | 37.3935 | Spain | 1980-1983 |
| Unger & Kurth 2010 | 50.4086 | Germany | 2000, 2005 |
| Unger & Kurth 2010 | 50.4272 | Germany | 2000 |
| Vogel unpublished | 47.2886 | Switzerland | 2003-2015 |
| Zimmermann 1951 | 47.3700 | Switzerland | 1949-50 |

**References (Table S1)**

Antikainen, E. (1978) *The Breeding Adaptation of the Jackdaw Corvus monedula L. in Finland.* Kuopio Naturalists' Society.

Antikainen, E. (1981) The Breeding Success of the Jackdaw *Corvus monedula* in Nesting Cells. *Ornis Fennica,* **58**, 72-77.

Argüello, C.R. & Alvarez, V.E. (1986) Fenologia y productividad de la grajilla (*Corvus monedula*) en la provincia de Leon. *Alytes,* **4**, 105-114.

Arnold, K.E. & Griffiths, R. (2003) Sex‐specific hatching order, growth rates and fledging success in jackdaws *Corvus monedula*. *Journal of Avian Biology,* **34**, 275-281.

Biondo, M. (1998) Intraspezifische Aggression, Populations- und Nahrungsökologie der Dohle *Corvus monedula* in Murten, Kanton Freiburg. *Der Ornithologischer Beobachter,* **95**, 203-220.

Chesney, M.C. (1986) The effects of poisoning by alpha-chloralose on the breeding success of a colony of Jackdaws *Corvus monedula* in Perthshire. *Bird Study,* **33**, 196-200.

Domínguez, L. (1999) *Ecología de la Grajilla (Corvus monedula Linnaeus 1758) en la provincia de Madrid.* PhD thesis, Universidad Complutense de Madrid.

Dwenger, R. (1989) *Die Dohle: corvus monedula.* Ziemsen.

Ejgelis, J.K. (1958) Feeding and economic importance of jackdaws in the oak forest of Vorskla. *Vestnik Leningradskogo Gosudarstvennogo Universiteta* **15**, 93-101.

Folk, C. (1968) Das Nisten und die Populationsdynamik der Dohle (*Corvus monedula* L.) in der CSSR. *Zoologicke Listy,* **17**, 221-236.

Freistaat Thuringen (2010) Dohlen in der Saaletalbrücke.

http://www.thueringen.de/th9/tlbv/abgeschlossene_bauprojekte/a4/teilprojekte/seite3/#

Gibbons, D.W. (1987) Hatching asynchrony reduces parental investment in the Jackdaw. *Journal of Animal Ecology,* **56**, 403-414.

Heeb, P. (1994) Intraclutch egg-mass variation and hatching asynchrony in the jackdaw (*Corvus monedula*). *Ardea,* **82**, 287-297.

Henderson, I.G. & Hart, P.J. (1993) Provisioning, parental investment and reproductive success in Jackdaws *Corvus monedula*. *Ornis Scandinavica,* **24**, 142-148.

Högstedt, G. (1977) Reduction of reproductive success in Jackdaws Corvus monedula breeding in a colony of Fieldfares Turdus pilaris. *Anser,* **16**, 143-145.

Hund, K. & Prinzinger, R. (1981) Notizen zur Brutbiologie der Elster *Pica pica,* Rabenkrache Corvus *Corone corone* und Dohle *Corvus monedula* in Wurttemberg. *Oekologie der Vögel*, **3**, 261-265.

Johnsson, K. (1994) Colonial breeding and nest predation in the jackdaw *Corvus monedula* using old black woodpecker *Dryocopus martius* holes. *Ibis,* **136**, 313-317.

Kamiński, P. (1989) Breeding ecology of a Jackdaw (*Corvus monedula*) colony nesting in natural holes in trees. *Nestling mortality of granivorous birds due to microorganisms and toxic substances* (eds Pinowski J., Kavanagh B.P. & Górski W.), pp. 84-98. Proceedings of International Symposium of the Working Group on Granivorous Birds, INTECOL, Słupsk, Poland.

Kamiński, P. & Konarzewski, M. (1984) Changes of body-weight, chemical-composition and energetic value in the nestlings of the jackdaw, *Corvus monedula* L., during their development in the nest. *Polish Journal of Ecology,* **32**, 125-139.

Lockie, J.D. (1955) The feeding and breeding of Jackdaws and Rooks with notes on Carrion Crows and other Corvidae. *Ibis,* **97**, 341-369.

Mayaud, N. (1933) Notes et remarques sur quelques corvidés. *Alauda,* **3**, 346-362.

Metz, E., Neitzsch, G. & Schindler, W. (1983) Die Dohle (*Corvus monedula*)- eine weitere bestandsgefährdete Vogelart in Hessen? *Vogel und Umwelt,* **2**, 313-319.

Pikula, J and Folk, C. (1970) Differential breeding in Corvus monedula, Sturnus vulgaris, Parus major and Fringilla coelebs in woodland and non-woodland habitats. *Zoologicke Listy,* **19**, 261-273.

Richford, AS. & Lawman D.M. (1978) The breeding of jackdaws on Skokholm. *Nature in Wales: the quarterly journal of the West Wales Field Society,* **16**, 110.

Riggenbach, H.E. (1979) Die Dohle *Corvus monedula* in der Schweiz. *Der Ornithologische Beobachter,* **76**, 153-168.

Röell, A. (1978) Social behaviour of the Jackdaw, *Corvus monedula*, in relation to its niche. *Behaviour,* **64**, 1 – 124.

Schmidt, K. (1988) Die Dohle (*Corvus monedula*) als Brutvogel im Bezirk Suhl (DDR) und erste Erfahrungen zum Schutz dieser gefahrdeten Vogelart. *Beihefte Veröffentlichungen Naturschutz Landschaftspflege Baden-Württemberg,* **53**, 191-210.

Schmidt, K. (2004) Vorkommen, Bestandssituation und Bruterfolg der Dohle *Corvus monedula* in Thuringen - Ergebnisse einer Bestandserfassung im Jahr 2002. *Anzeiger des Vereins Thüringer Ornithologen,* **3**, 67-76.

Schmidt, K. & Schmidt, M. Zum (1994) Vorkommen und zur Brutbiologie der Dohle (*Corvus monedula*) in Süd-thiiringen. *Naturschutzreport,* **7**, 326-336.

Schoof, E. (1977) Die Bad Wildunger Dohlen Bestandsentwicklung und Verhaltensbeobachtungen. *Vogelkundliche Hefte,* **3**, 37-50.

Soler, M. (1984) *Biometría y biología de la Grajilla (Corvus monedula L.).* PhD thesis, Universidad de Granada.

Soler, M. (1990) Breeding success and productivity in the Jackdaw (*Corvus monedula* L.) in Granada (Spain). *Granivorous bird in the agricoltural landscape* (eds Pinowski J. & Summers-Smith J.D.), pp. 253-261. Proceedings of International Symposium of the Working Group on Granivorous Birds, INTECOL, Ottawa, Canada.

Strebel, S. (1991) Bruterfolg und dessen Kausalfaktoren bei der Dohle *Corvus monedula* in der Kolonie Murten FR. PhD thesis, University of Bern.

Unger, C. & Kurth, K. (2010) Untersuchungen zur Brutbiologie und zur Habitatwahl bei der Dohle *Coloeus monedula* im Landkreis Hildburghausen, Siidthuringen. *Anzeiger des Vereins Thüringer Ornithologen,* **7**, 95-107.

Zimmermann, D. (1951) Zur brutbiologie der Dhole, *Corvus monedula* (L.). *Der Ornithologischer Beobachter,* **48**, 73-111.

**Table S2: Determinants of clutch size and number of fledglings per breeding pair across Europe.** The reference category is “Agricultural”. *Agricultural* = breeding site in agricultural area, *Urban* = breeding site in urban area with agricultural foraging grounds within 100 m (*UrbanC*)*,* within 100 - 500 m (*UrbanI),* further than 500 m away (*UrbanF)*, *Wood* = breeding site in woodland. Rainfall is expressed as average millimetres of rainfall per day for a given month, year and site. Significant results are presented in bold. CrI corresponds to the 95% Bayesian Credible Interval.

|  |  |  | **Clutch size$** | |  | **Number of fledglings per breeding pair$** | |
| --- | --- | --- | --- | --- | --- | --- | --- |
| **Effect** | |  | **Estimate** | **CrI** |  | **Estimate** | **CrI** |
| Intercept | |  | 5.16 | 4.46; 5.86 |  | 3.15 | 2.13; 4.22 |
| Breeding habitat | |  |  |  |  |  |  |
|  | Agricultural (N=80) |  | / | / |  | / | / |
|  | UrbanC (N=36) |  | 0.38 | -0.17; 0.93 |  | 0.21 | -0.34; 0.74 |
|  | UrbanI (N=80) |  | 0.22 | -0.26; 0.73 |  | **-0.37** | **-0.69; -0.12** |
|  | UrbanF (N=39) |  | 0.16 | -0.29; 0.60 |  | **-0.62** | **-1.07; -0.19** |
|  | Wood (N=41) |  | -0.26 | -0.75; 0.22 |  | **-0.79** | **-1.38; -0.25** |
| log(colony size) | |  | **-0.14** | **-0.27; -7.14E-03** |  | **-0.34** | **-0.52; -0.15** |
| Latitude | |  | **-0.02** | **-0.04; -8.52E-04** |  | **0.04** | **0.02; 0.08** |
| Latitude^2^ | |  | 8.83E-06 | -1.96E-03; 2.11E-03 |  | -3.83E-03 | **-7.72E-03; -3.82E-04** |
| Rainfall April (mm) | |  | -0.10 | -0.33; 0.13 |  | - | - |
| Rainfall April ^2^ (mm) | |  | 0.01 | -0.04; 0.05 |  | - | - |
| Rainfall May (mm) | |  | - | - |  | 0.17 | -0.24; 0.53 |
| Rainfall May ^2^ (mm) | |  | - | - |  | -0.03 | -0.10; 0.05 |
| Rainfall June (mm) | |  | - | - |  | -0.05 | -0.44; 0.33 |
| Rainfall June ^2^ (mm) | |  | - | - |  | 0.01 | -0.05; 0.07 |
| *$ distribution = lognormal; link function = identity; random factors =* *source, site and year.*  - = parameter not tested in the given model. | | | | | | | |

**Appendix S3: Range use of GPS-logged jackdaws in Murten and Galmiz/Kerzers**

During the breeding season 2013, we equipped six breeding adults in Murten and six in Galmiz/Kerzers with solar-powered GPS loggers fixed with a backpack-style harness (one pair and four single birds equipped with the same logger in each colony). These loggers were developed and manufactured at the Workshops for Scientific Support and Equipment of the University of Konstanz, Germany (weight: 12 - 16 g and dimensions: 4 x 2 x 1.5 cm). The weight of the loggers corresponds to about 6% of the mean weight of the jackdaws caught (231 g), which is slightly above the rule of thumb (5%) but according to Barron, Brawn & Weatherhead (2010) in the range of acceptance. The data could be downloaded remotely from about 100 m. Observations on the days following the fixation showed that jackdaws with loggers behaved normally, as far as we could observe. Their breeding behaviour was absolutely comparable to non-loggered conspecifics. Therefore, we consider the results of the size of the home range as representative and comparable to other telemetry studies. Permission for putting GPS loggers was given by the Service de la sécurité alimentaire et des affaires vétérinaires SAAV of the Canton of Fribourg and the Federal Office for the Environment. This technique was used to investigate whether both populations (urban and agricultural) varied in the distance from the nest at which they foraged and whether birds of the urban colony (Murten) foraged within the city limits or beyond. These loggers sampled the position every 8 or 30 minutes if the battery load was high, otherwise every hour (depending on the programming). Several loggers had an energy-saving shutdown function for darkness and a smaller battery to reduce weight (equally distributed between colonies). Data collection was running for one year. Finally for the postbreeding period we have data from 2013 (eight individuals, 1061 fixes) and for the prebreeding period from 2014 (two individuals, 265 fixes), while for the breeding period we have data from both years (nine individuals, 1285 fixes). The low “return-rate” was at least partially due to logger-failure, as several (in each colony at least 2 individuals) birds with loggers have been observed without transmitting any data. Otherwise we cannot differentiate between logger failure, permanent emigration out of the study area or death.

The three periods were defined as follows: the prebreeding period corresponded to the 30 days before egg laying which included nest building; the breeding period was from laying of the first egg until chick fledging (approx. 55 days); the postbreeding period consisted of the 30 days after fledging. For each period, kernel density estimates (75% and 95% CrI) of the area used were computed in R (R Core Team 2015) with the adehabitatHR package (Calenge, C. 2006) and the smoothing parameter *h* was set to *href*. All data within each period were pooled (Otis & White 1999), as we were interested only in the overall view of the area used by jackdaws breeding in Murten and in Galmiz/Kerzers, respectively. The computed kernel utilisation distributions were then imported into QGIS 2.12.2-Lyon for creating the maps (Fig. S3a, b, c). The range used during the prebreeding and breeding period by jackdaws from Murten and Galmiz/Kerzers do not overlap, while they overlap during the postbreeding period.

We analysed statistically whether the distances between GPS locations and breeding sites differed depending on the breeding population (Murten vs. Galmiz/Kerzers) and between the periods (prebreeding, breeding, or postbreeding). For this, we used a generalized linear mixed model where the response variable was the distances (log-transformed) and the explanatory variables were *Site, Period* and the interaction of both. We accounted for *Year* and *Individual* as random factors (Table S3). This model was performed with the function *lmer* from package *lme4* (Bates et al. 2012) and using the Bayesian framework. We simulated a random sample (N = 5000) from the joint posterior distribution of the model parameters using the function sim from package arm (Gelman & Hill 2006). From this sample, we used the 2.5% and 97.5% quantiles as lower and upper limit of the 95% credible interval (CrI), and an effect was considered significant when the 95% CrI did not contain zero.

**References (Appendix S3)**

Bates D, Maechler M, Bolker B, and Walker S. 2012. lme4: Linear mixed-effects models using S4 classes. *R Packag version 0999999-0*.

Barron, D., Brawn, J. & Weatherhead, P. (2010). Meta analysis of transmitter effects on avian behaviour and ecology. *Methods in Ecology and Evolution*, **1**, 180-187.

Calenge, C. (2006) The package adehabitat for the R software: a tool for the analysis of space and habitat use by animals. *Ecological Modelling*, **197**, 516-519

Gelman, A. & Hill, J. 2006. Data Analysis using Regression and Multilevel/Hierarchical Models. New York: Cambridge University Press.

Otis, D. L. & White, G. C. (1999). Autocorrelation of location estimates and the analysis of radiotracking data. *The Journal of Wildlife Management*, **63**, 1039-1044

Quantum GIS Development Team (2015) Version 2.12.2. Quantum GIS geographic information system. Open Source Geospatial Foundation Project. Available at: http://qgis.osgeo.org

R Development Core Team. (2015) R: A language and environment for statistical computing. R Foundation for Statistical Computing, Vienna, Austria.


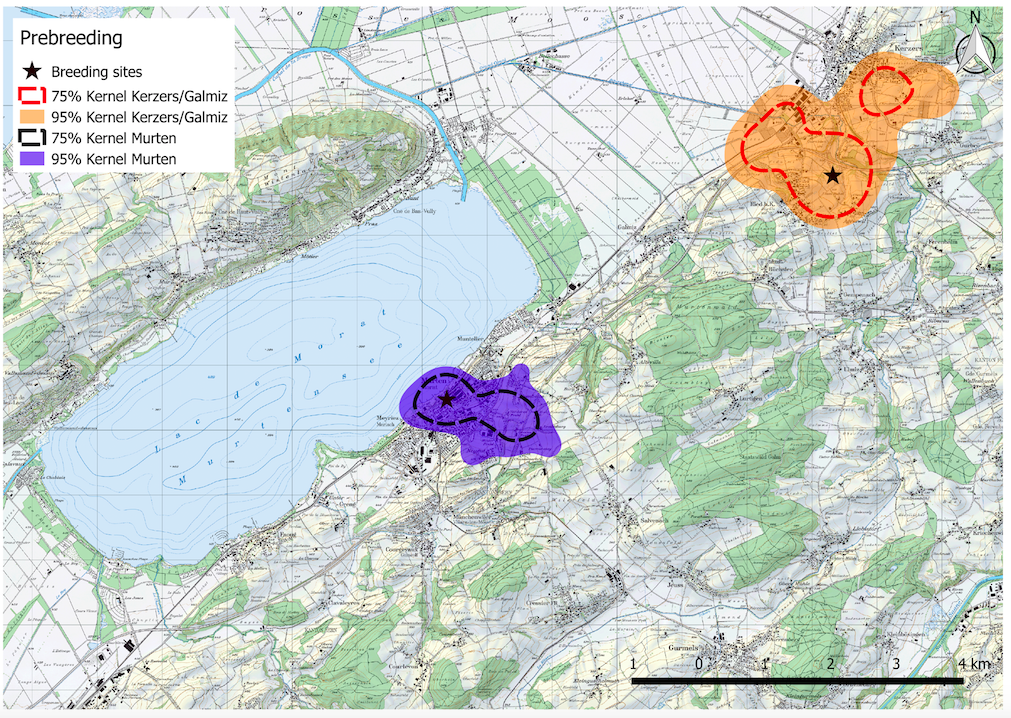

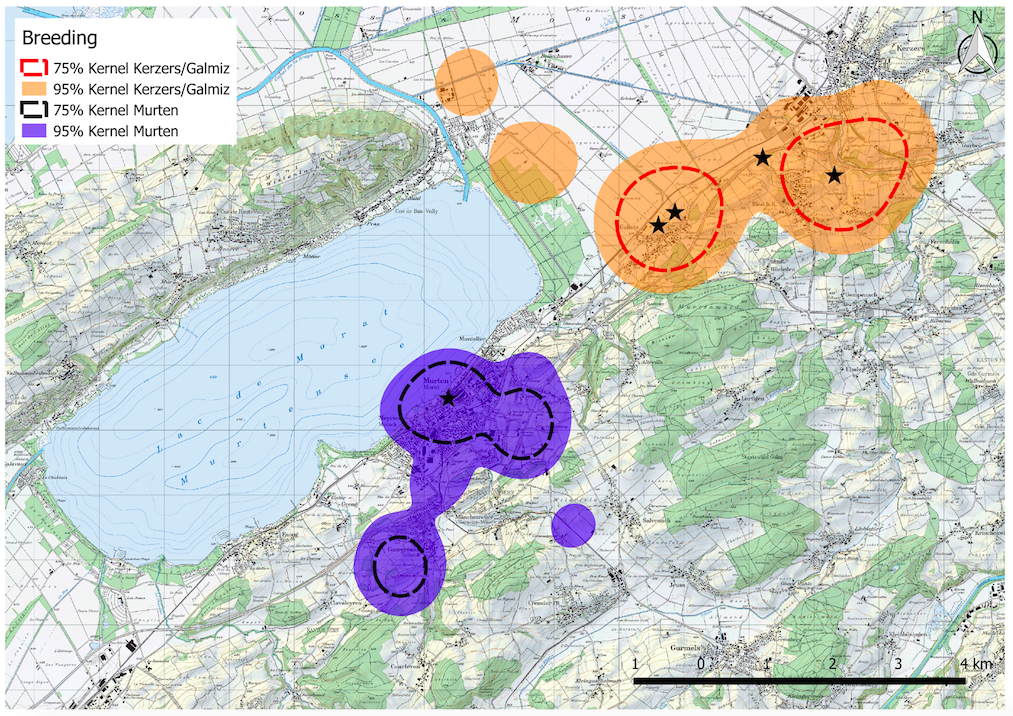

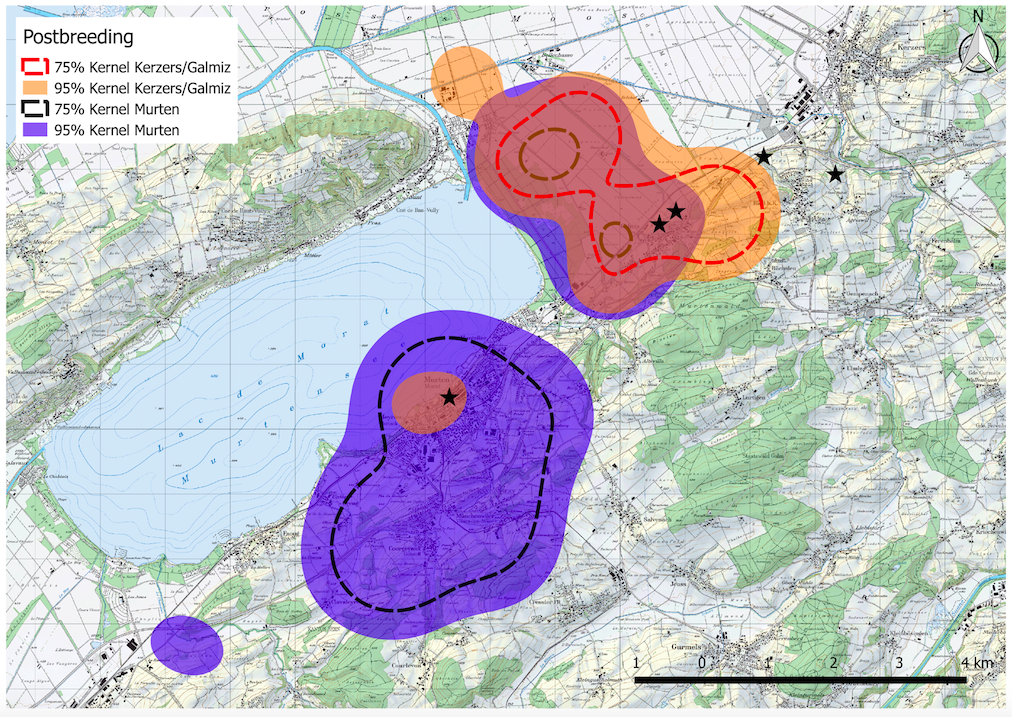


a)

b)

c)

**Figure S4. Map of the study area during the three periods of the breeding season.** a) Pre-breeding period (30 days before laying), b) breeding period (from laying to chick fledgling, around 55 days) and c) Post-breeding period (30 days after chick fledgling).

Reproduced by permission of swisstopo (BA16034). Stars depict the breeding sites where the adults had been caught.

**Table S5. Determinants of the distance travelled by jackdaws of Murten and Galmiz/Kerzers** from their nest during the prebreeding, breeding and postbreeding periods. The reference categories are Galmiz/Kerzers and the prebreeding period. Significant results are presented in bold. CrI is the 95% Bayesian Credible Interval.

|  |  |  | **Distance travelled ^$^** | |
| --- | --- | --- | --- | --- |
| **Effect** | |  | **Estimate** | **CrI** |
| Intercept | |  | 5.08 | 4.00; 6.17 |
| Site | |  |  |  |
|  | Galmiz/Kerzers |  | / | / |
|  | Murten |  | - 0.47 | -1.74; 0.77 |
| Periods | |  |  |  |
|  | Prebreeding |  | / | / |
|  | Breeding |  | **- 0.36** | **-0.64; -0.06** |
|  | Postbreeding |  | **1.76** | **1.41; 2.11** |
| Murten:Breeding | |  | **0.80** | **0.37; 1.25** |
| Murten:Postbreeding | |  | 0.20 | -0.36; 0.75 |
| *^$^ distribution = lognormal; link function = identity; random factor = years and individuals* | | | | |


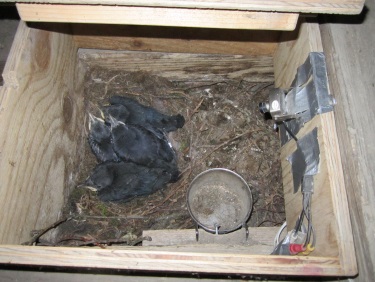


**Figure S6. Photograph of a nestbox in the Murten (urban) colony.** The arrow indicates the exit of the nest box and the red circle the feeder.

**Table S7: Comparison of egg volume from nests food supplemented during prebreeding and egg laying (U_Fed) with those of urban control nests (U_Con) and of agricultural nests (A_Con) (experiment 1; 2014).** The reference category is the urban control pairs. Significant results are presented in bold. CrI is the 95% Bayesian Credible Interval.

|  |  |  | **Clutch size* (N=24 nests)** | |  | **Egg volume ** (N=109 eggs)** | |  | **Incubation duration ^$^ (N=12 nests)** | |  |
| --- | --- | --- | --- | --- | --- | --- | --- | --- | --- | --- | --- |
|  | **Terms** | | **Estimate** | **CrI** |  | **Estimate** | **CrI** |  | **Estimate** | **CrI** |  |
|  | Intercept | | 6.37 | -6.70; 19.12 |  | 5.19E03 | -2.24E04; 3.14E04 |  | 2.76 | 1.97; 3.58 |  |
|  | Food treatment in 2014 | |  |  |  |  |  |  |  |  |  |
|  |  | U_Con (n=8) | / | / |  | / | / |  | / | / |  |
|  |  | U_Fed (n=7) | 0.04 | -0.99; 1.08 |  | **1.45E03** | **3.32E02; 2.52E03** |  | -0.03 | -0.08; 0.05 |  |
|  |  | A_Con (n=9) | 0.23 | -1.02; 1.49 |  | **1.39E03** | **6.75E01; 2.75E03** |  | -5.91E-03 | -0.06; 0.02 |  |
|  | Clutch size | | / | / |  | 3.44E02 | -1.99E02; 8.80E02 |  | 0.01 | -0.01; 0.04 |  |
|  | Laying date | | -0.02 | -0.35; 0.32 |  | 1.08E02 | -1.97E02; 4.14E02 |  | -1.78E-03 | -7.99E-03; 3.99E-03 |  |
|  | Mean Temperature (ºC) † | | 0.11 | -1.78; 1.84 |  | -6.60E02 | -2.35E03; 9.99E03 |  | 0.02 | -0.04; 0.08 |  |
|  | Number of rainy † | | / | / |  | 1.17E02 | -1.24E03; 1.44E03 |  | / | / |  |
|  | Number of rainy days ^2^ † | | / | / |  | -2.27E01 | -9.19E01; 4.71E01 |  | / | / |  |
| ** Distribution * normal; link function = identity.*  *** Distribution ** normal; link function = identity; random factor = nestbox_ID + brood_ID*  *$ Distribution l* $ *lognormal; link function = identity; random factor = nestbox_ID*  *† weather data I* † *weather data is specific for each period ( model) and nest.* | | | | | | | | | | | |

*T****able S8: Determinants of nestlings weight from nests food supplemented during prebreeding and egg laying (U_Fed) with those of urban control nests (U_Con) and of agricultural control nests (A_Con) (experiment 1; 2014).*** *The reference category is the urban control pairs. Significant results are presented in bold. CrI is the 95% Bayesian Credible Interval.*

|  |  | **Hatchling weight * (N=84 chicks)** | |  | **Fledgling weight * (N=44 chicks)** | |
| --- | --- | --- | --- | --- | --- | --- |
| **Effects** | | **Estimate** | **CrI** |  | **Estimate** | **CrI** |
| Intercept | | 5.97 | -19.91; 31.20 |  | -3.04 | -6.44E02; 6.49E02 |
| Food treatment in 2014 | |  |  |  |  |  |
|  | U_Con (n=8) | / | / |  | / | / |
|  | U_Fed (n=7) | 0.59 | -0.42; 1.62 |  | -17.75 | -64.51; 29.20 |
|  | A_Con (n=9) | 0.22 | -0.84; 1.33 |  | 5.71 | -35.92; 47.13 |
| Hatching date | | **-0.16** | **-0.28; -0.04** |  | 1.34 | -3.89; 6.44 |
| Mean Temperature (°C) † | | 0.61 | -0.61; 1.85 |  | ¶ | ¶ |
| Number of rainy †† | | 2.45 | -0.88; 5.81 |  | 4.75 | -18.15; 27.36 |
| Number of rainy days^2^ †† | | -0.09 | -0.21; 0.04 |  | 0.47 | -3.37; 4.19 |

** distribution = normal; link function = identity.*

† *weather data is specific for each period ( model) and nest.*

¶ *variable correlated with hatching date, removed from analysis*

**Table S9: Comparison of hatching success, chick survival, as well as the feeding frequency of the urban nests food supplemented during pre-breeding and egg-laying (U_Fed) with those of urban control nests (U_Con) and agricultural nests (A_Con) in 2014.** The reference category is the urban control pairs. Significant results are presented in bold. CrI is the 95% Bayesian Credible Interval.

|  |  | **Hatching success ***  **(N=24 nests)** | |  | **Survival at 5 days * (N=24 nests)** | |  | **Survival at 10 days * (N=24 nests)** | |  | **Survival at 17 days * (N=24 nests)** | |  | **Survival at 27 days * (N=24 nests)** | |  | **Feeding frequency ^$^**  **(N=689 fixes)** | |
| --- | --- | --- | --- | --- | --- | --- | --- | --- | --- | --- | --- | --- | --- | --- | --- | --- | --- | --- |
| **Effects** | | **Estimate** | **CrI** |  | **Estimate** | **CrI** |  | **Estimate** | **CrI** |  | **Estimate** | **CrI** |  | **Estimate** | **CrI** |  | **Estimate** | **CrI** |
| Intercept | | 2.88 | -6.03; 11.89 |  | 15.86 | 4.11; 27.36 |  | 19.30 | 7.99; 30.98 |  | 12.94 | 1.96; 23.37 |  | 10.63 | 0.25; 21.21 |  | 0.7 | 0.3; 1.1 |
| Food treatment in 2014 | |  |  |  |  |  |  |  |  |  |  |  |  |  |  |  |  |  |
|  | U_Con (n=8) | / | / |  | / | / |  | / | / |  | / | / |  | / | / |  | / | / |
|  | U_Fed (n=7) | **0.79** | **0.06; 1.66** |  | -0.62 | -2.04; 0.83 |  | -1.57 | -3.06; 0.09 |  | **-1.24** | **-2.61; -0.09** |  | **-1.5** | **-2.85; -0.23** |  | -2.34E-03 | -0.35; 0.34 |
|  | A_Con (n=9) | **1.77** | **0.76; 2.92** |  | 0.70 | -0.90; 2.28 |  | -0.15 | -1.54; 1.31 |  | 0.59 | -0.95; 2.11 |  | 0.17 | -1.16; 1.47 |  | -0.11 | -0.46; 0.24 |
| Hatching date | | 0.04 | -0.04; 0.14 |  | **-0.14** | **-0.28; -0.01** |  | **-0.15** | **-0.23; -0.06** |  | **-0.09** | **-0.35; -0.01** |  | **-0.08** | **-0.47; -0.02** |  | - | - |
| Chicks age | | - | - |  | - | **-** |  | - | - |  | - | - |  | - | - |  | **0.05** | **0.03; 0.07** |
| Chicks age ^2^ | | - | - |  | - | **-** |  | - | - |  | - | - |  | - | - |  | **-2.15E-03** | **-2.83E-03; -1.51E-03** |
| Log(Brood size) | | - | - |  | - | **-** |  | - | - |  | - | - |  | - | - |  | 0.33 | **0.10; 0.57** |
| Mean Temperature (°C) † | | -0.78 | -1.47; 0.07 |  | 0.09 | -0.28; 0.47 |  | ¶ | ¶ |  | ¶ | ¶ |  | ¶ | ¶ |  | -0.01 | -0.02; 0.01 |
| Rainfall per day (mm) | | - | - |  | - | **-** |  | - | - |  | - | - |  | - | - |  | 2.25E-04 | -0.01; 0.01 |
| ** distribution = normal; link function = identity.*  *$ distribution = poisson; link function = log; random factor = brood_ID + date*  ¶ *variable correlated with hatching date, removed from analysis*  *† mean temperature is specific for each nest as well as each period (model) for hatching success and survival whereas it is specific to the day for the feeding frequency model*  *††* *number of rainy day are specific for each period (model) and nest.*  *- = parameter not tested in the given model* | | | | | | | | | | | | | | | | | | |

**Table S10: Determinants of clutch size, eggs parameters, and duration of incubation** **of jackdaw urban nests.** U_Fed = in 2014 fed until egg laying and in 2015 fed until chick fledging. U_Con = urban control nests in both 2014 and 2015**.** The reference categories are the urban control pairs and the year 2014. Significant results are presented in bold. CrI is the 95% Bayesian Credible Interval.

|  |  | **Clutch size* (N=34 nests)** | |  | | **Egg volume ** (N=161 eggs)** | | |  | | **Egg weight ** (N=161 eggs)** | | |  | **Incubation duration $ (N=26 nests)** | | | | |  | | |
| --- | --- | --- | --- | --- | --- | --- | --- | --- | --- | --- | --- | --- | --- | --- | --- | --- | --- | --- | --- | --- | --- | --- |
| **Effects** | | **Estimate** | **CrI** |  | | **Estimate** | **CrI** | |  | | **Estimate** | **CrI** | |  | **Estimate** | | | **CrI** | |  | | |
| Intercept | | 4.87 | 3.97; 5.72 |  | | 9.68E03 | 8.27E03; 1.11E04 | |  | | 11.36 | 10.62; 12.12 | |  | 3.04 | | | 2.40; 3.63 | |  | | |
| Food treatment (both years pooled) | | |  |  | |  |  | |  | |  |  | |  |  | | |  | |  | | |
|  | U_Con (n=17) | / | / |  | | / | / | / | | / | | | / | | |  | / | | / | |  | |
|  | U_Fed (n=17) | 0.02 | -0.77; 0.78 |  | | **1.49E03** | **8.73E02; 2.1E03** | |  | | **1.49** | **0.65; 2.29** | |  | **-0.05** | | | **-0.10; -0.01** | |  | | |
| Year | |  |  |  |  | |  | |  | |  |  | |  |  | | |  | |  | | |
|  | 2014 (n=15) | / | / |  | | / | / | |  | | / | / | |  | / | | | / | |  | | |
|  | 2015 (n=19) | -0.53 | -1.50; 0.50 |  | | **6.98E02** | **5.5E01; 1.36E03** | |  | | **0.94** | **0.29; 1.60** | |  | -0.05 | | | -0.09; 0.01 | |  | | |
| Food treatment:Year | | Fig. 4 | Fig. 4 |  | **Fig. 4** | | **Fig. 4** | |  | | **Fig. 4** | **Fig. 4** | |  | Fig. 4 | | | Fig. 4 | |  | | |
| Clutch size | | - | - |  | 1.84E02 | | -6.95E01; 4.39E02 | |  | | 0.26 | -0.02; 0.52 | |  | 2.04E-2 | | | -4.61E-07; 3.97E-02 | |  | | |
| Laying date | | -0.04 | -0.29; 0.20 |  | **-2.48E02** | | **-4.11E02; -8.33E01** | |  | | **-0.23** | **-0.43; -0.02** | |  | -1.64E-03 | | | -7.48E-03; 3.69E-03 | |  | | |
| Mean Temperature (°C) † | | 0.55 | -0.64; 1.76 |  | **8.61E02** | | **6.86E01; 1.63E03** | |  | | 0.88 | -0.07; 1.88 | |  | -0.01 | | | -0.05; 0.03 | |  | | |
| Number of rainy days † | | / | / |  | 2.55E02 | | -6.83E01; 5.74E02 | |  | | 0.19 | -0.11; 0.59 | |  | / | | | / | |  | | |
| Number of rainy days ^2^ † | | / | / |  | -2.11E01 | | -5.68E01; 1.42E01 | |  | | -0.01 | -0.06; 0.03 | |  | / | | | / | |  | | |
| ** distribution = normal; link function = identity; random factor = nestbox_ID*  *** distribution = normal; link function = identity; random factor = nestbox_ID + brood_ID*  *$ distribution lognormal; link function = identity; random factor = nestbox_ID*  *† weather data is specific for each period ( model) and nest.* | | | | | | | | | | | | | | | | | | | | | |  |

**Table S11: Comparisons of nestling weight at hatching and the different age categories between fed and unfed groups. U_Fed; in 2014 fed until egg laying and in 2015 fed until chick fledging and urban control pairs (U_Con; in 2014 and 2015). The reference categories are the urban control pairs and the year 2014. Significant results are presented in bold. CrI is the 95% Bayesian Credible Interval.**

|  |  | **Hatchling weight***  **(N=99 chicks)** | |  | **Weight at 5 days ***  **(N=62 chicks)** | |  | **Weight at 10 days ***  **(N=54 chicks)** | | |  | **Weight at 17 days ***  **(N=47 chicks)** | | |  | **Weight at 27 days ***  **(N=41 chicks)** | | |  | |  |
| --- | --- | --- | --- | --- | --- | --- | --- | --- | --- | --- | --- | --- | --- | --- | --- | --- | --- | --- | --- | --- | --- |
| **Effects** | | **Estimate** | **CrI** |  | **Estimate** | **CrI** |  | | **Estimate** | **CrI** |  | | **Estimate** | **CrI** |  | | **Estimate** | **CrI** | |  | |
| Intercept | | 9.03 | 8.28; 9.77 |  | 54.8 | 42.1; 68.3 |  | | 93.2 | 72.9; 113.3 |  | | 175.3 | 141.5; 210.1 |  | | 192.4 | 162.2; 222.7 | |  | |
| Food treatment (both years pooled) | |  |  |  |  |  |  | |  |  |  | |  |  |  | |  |  | |  | |
|  | U_Con (N=17) | / | / |  | / | / |  | | / | / |  | | / | / |  | | / | / | |  | |
|  | U_Fed (n=17) | **1.26** | **0.2; 2.32** |  | -6.5 | -19.92; 7.41 |  | | -8.66 | -39.4; 21.5 |  | | -16.8 | -52.5; 22.9 |  | | -9.09 | -45.9; 30.5 | |  | |
| Year | |  |  |  |  |  |  | |  |  |  | |  |  |  | |  |  | |  | |
|  | 2014 (n=15) | / | / |  | / | / |  | | / | / |  | | / | / |  | | / | / | |  | |
|  | 2015 (n=19) | 0.75 | -0.2; 1.7 |  | -5.63 | -19.1; 8.33 |  | | 25.6 | -4.61; 52.4 |  | | 1.86 | -39.9; 41.5 |  | | 29.5 | -4.12; 62.6 | |  | |
| Food treatment:Year | | **Fig. 4** | **Fig. 4** |  | Fig. 4 | Fig. 4 |  | | Fig. 4 | Fig. 4 |  | | Fig. 4 | Fig. 4 |  | | Fig. 4 | Fig. 4 | |  | |
| Hatching date | | -0.07 | -0.15; 0.01 |  | -0.94 | -1.94; 0.05 |  | | -0.25 | -2.53; 2.00 |  | | -3.37 | -8.54; 0.38 |  | | 2.31 | -1.58; 6.03 | |  | |
| Mean Temperature (°C) † | | ¶ | ¶ |  | -0.11 | -2.28; 2.38 |  | | -1.87 | -12.5; 8.95 |  | | ¶ | ¶ |  | | ¶ | ¶ | |  | |
| Number of rainy †† | | -0.22 | -0.53; 0.09 |  | 4.01 | -2.15; 9.56 |  | | -5.74 | -22.1; 10.22 |  | | -17.9 | -38.5; 2.02 |  | | 2.44 | -2.53; 6.94 | |  | |
| Number of rainy days ^2^ †† | | 0.03 | -0.1; 0.17 |  | -5.37 | -12.7; 2.37 |  | | 5.82 | -6.55; 17.8 |  | | -9.65 | -20.31; 0.96 |  | | -0.72 | -2.00; 0.57 | |  | |
| ** distribution = normal; link function = identity; random factor = nestbox_ID*  ¶ *variable correlated with hatching date, removed from analysis*  *† mean temperature is specific for each nest as well as each period (model) for hatching success and survival whereas it is specific to the day for the feeding frequency model*  *†† number of rainy day are specific for each period (model) and nest.* | | | | | | | | | | | | | | | | | | | | | |

**Table S12: Comparison of hatching success, nestling survival, and feeding frequency of fed nests (U_Fed; in 2014 fed until egg laying and in 2015 fed until chick fledging) and urban control pairs (U_Con; in 2014 and 2015).** The reference categories are the urban control pairs and the year 2014. Significant results are presented in bold. CrI is the 95% Bayesian Credible Interval.

|  |  | **Hatching success ***  **(N=34 nests)** | |  | **Survival 5 days ***  **(N=34 nests)** | |  | **Survival 10 days ***  **(N=34 nests)** | | |  | **Survival 17 days ***  **(N=34 nests)** | | |  | **Survival 27 days ***  **(N=34 nests)** | | |  | | **Feeding frequency $**  **(N=269 fixes)** | | |  | |
| --- | --- | --- | --- | --- | --- | --- | --- | --- | --- | --- | --- | --- | --- | --- | --- | --- | --- | --- | --- | --- | --- | --- | --- | --- | --- |
| **Effects** | | **Estimate** | **CrI** |  | **Estimate** | **CrI** |  | | **Estimate** | **CrI** |  | | **Estimate** | **CrI** |  | | **Estimate** | **CrI** | |  | | **Estimate** | **CrI** | |  |
| Intercept | | 0.05 | -1.07; 1.22 |  | 0.66 | -0.66; 2.20 |  | | 0.81 | -0.30; 1.94 |  | | 0.28 | -1.07; 1.67 |  | | 0.70 | -0.56; 2.01 | |  | | 0.62 | 0.01; 1.26 | |  |
| Food treatment (both years pooled) | |  |  |  |  |  |  | |  |  |  | |  |  |  | |  |  | |  | |  |  | |  |
|  | U_Con (N=17) | / | / |  | / | / |  | | / | / |  | | / | / |  | | / | / | |  | | / | / | |  |
|  | U_Fed (n=17) | **1.43** | **0.13; 2.96** |  | -0.69 | -2.05; 0.65 |  | | **-1.27** | **-2.37; -0.12** |  | | **-1.38** | **-2.98; -0.15** |  | | **-1.74** | **-3.28; -0.23** | |  | | -0.21 | -0.55; 0.12 | |  |
| Year | |  |  |  |  |  |  | |  |  |  | |  |  |  | |  |  | |  | |  |  | |  |
|  | 2014 (n=15) | / | / |  | / | / |  | | / | / |  | | / | / |  | | / | / | |  | | / | / | |  |
|  | 2015 (n=19) | **2.45** | **0.56; 4.28** |  | -0.47 | -2.06; 0.99 |  | | -0.9 | -2.27; 0.39 |  | | -0.5 | -2.11; 1.09 |  | | -0.23 | -1.83; 1.33 | |  | | **-0.35** | **-0.71; -0.01** | |  |
| Food treatment:Year | | Fig. 4 | Fig. 4 |  | **Fig. 4** | **Fig. 4** |  | | **Fig. 4** | **Fig. 4** |  | | **Fig. 4** | **Fig. 4** |  | | Fig. 4 | Fig. 4 | |  | | 0.27 | -0.2; 0.76 | |  |
| Hatching date | | -0.02 | -0.14; 0.10 |  | -0.08 | -0.15; 0.01 |  | | -0.04 | -0.15; 0.06 |  | | -0.05 | -0.20; 0.10 |  | | -0.01 | -0.09; 0.24 | |  | | 0.07 | -0.09; 0.24 | |  |
| Chicks age | | - | - |  | - | **-** |  | | - | - |  | | - | - |  | | - | - | |  | | -1.78E-03 | -0.07; 0.07 | |  |
| Chicks age ^2^ | | - | - |  | - | **-** |  | | - | - |  | | - | - |  | | - | - | |  | | -1.31E-04 | -3.12E-03; 2.70E-03 | |  |
| Log(Brood size) | | - | - |  | - | **-** |  | | - | - |  | | - | - |  | | - | - | |  | | **0.47** | **0.11; 0.83** | |  |
| Mean Temperature (°C) † | | ¶ | ¶ |  | -0.09 | -0.30; 0.14 |  | | -0.08 | -0.37; 0.70 |  | | ¶ | ¶ |  | | ¶ | ¶ | |  | | 0.01 | -0.01; 0.03 | |  |
| Rainfall per day (mm) | | - | - |  | - | **-** |  | | - | - |  | | - | - |  | | - | - | |  | | 0.01 | -0.01; 0.03 | |  |
| ** distribution = binomial; link function = logit; random factor = nestbox_ID*  *$ distribution = poisson; link function = identity; random factor = brood_ID + nestbox_ID + date*  ¶ *variable correlated with hatching date, removed from analysis*  *† mean temperature is specific for each nest as well as each period (model) for hatching success and survival whereas it is specific to the day for the feeding frequency model*  *- = parameter not tested in the given model.* | | | | | | | | | | | | | | | | | | | | | | | | | |
